# Transplanted human p75-positive stem Leydig cells replace disrupted Leydig cells for testosterone production

**DOI:** 10.1038/cddis.2017.531

**Published:** 2017-10-12

**Authors:** Min Zhang, Jiancheng Wang, Chunhua Deng, Mei Hua Jiang, Xin Feng, Kai Xia, Weiqiang Li, Xingqiang Lai, Haipeng Xiao, Ren-shan Ge, Yong Gao, Andy Peng Xiang

**Affiliations:** 1 Department of Andrology, The First Affiliated Hospital, Sun Yat-sen University, Guangzhou, China; 2 Center for Stem Cell Biology and Tissue Engineering, Key Laboratory for Stem Cells and Tissue Engineering, Ministry of Education, Sun Yat-sen University, Guangzhou, China; 3 Guangdong Provincial Key laboratory of Orthopedics and Traumatology, Guangzhou, China; 4 Department of Anatomy and Neurobiology, Zhongshan School of Medicine, Sun Yat-sen University, Guangzhou, China; 5 Department of Biochemistry, Zhongshan School of Medicine, Sun Yat-sen University, Guangzhou, China; 6 Department of Endocrinology, The First Affiliated Hospital of Sun Yat-sen University, Guangzhou, China; 7 The Second Affiliated Hospital and Yuying Children's Hospital of Wenzhou Medical University, Wenzhou, Zhejiang, China; 8 Reproductive Medicine Center, The Key Laboratory for Reproductive Medicine of Guangdong Province, The First Affiliated Hospital of Sun Yat-sen University, Wenzhou, Guangzhou, China; 9 Key Laboratory of Protein Modification and Degradation, School of Basic Medical Sciences, Affiliated Cancer Hospital and Institute of Guangzhou Medical University, Guangzhou, China

## Abstract

Previous studies have demonstrated that rodent stem Leydig cell (SLC) transplantation can partially restore testosterone production in Leydig cell (LC)-disrupted or senescent animal models, which provides a promising approach for the treatment of hypogonadism. Here, we isolated human SLCs prospectively and explored the potential therapeutic benefits of human SLC transplantation for hypogonadism treatment. In adult human testes, p75 neurotrophin receptor positive (p75^+^) cells expressed the known SLC marker nestin, but not the LC lineage marker hydroxysteroid dehydrogenase-3*β* (HSD3*β*). The p75^+^ cells which were sorted by flow cytometry from human adult testes could expand *in vitro* and exhibited clonogenic self-renewal capacity. The p75^+^ cells had multi-lineage differentiation potential into multiple mesodermal cell lineages and testosterone-producing LCs *in vitro*. After transplantation into the testes of ethane dimethane sulfonate (EDS)-treated LC-disrupted rat models, the p75^+^ cells differentiated into LCs *in vivo* and secreted testosterone in a physiological pattern. Moreover, p75^+^ cell transplantation accelerated the recovery of serum testosterone levels, spermatogenesis and reproductive organ weights. Taken together, we reported a method for the identification and isolation of human SLCs on the basis of p75 expression, and demonstrated that transplanted human p75^+^ SLCs could replace disrupted LCs for testosterone production. These findings provide the groundwork for further clinical application of human SLCs for hypogonadism.

Male hypogonadism is a symptomatic clinical syndrome caused by testosterone deficiency, which is characterized by sexual dysfunction, osteoporosis, amyotrophy, central adiposity and others.^1^ In 2010, the European Male Ageing Study reported that 17.0% of men aged 40–79 years had serum testosterone levels below the normal values, indicating the high prevalence of hypogonadism among middle-aged and elderly males.^2^ Epidemiological studies suggest that hypogonadism not only adversely affects patients' quality of life, but also increases the risk of diabetes,^3^ arteriosclerosis^4^ and dementia.^5^ Exogenous testosterone supplementation has some beneficial effects, including the improvement of sexual function, muscle mass, bone density and body composition.^6, 7^ However, it disrupts the hypothalamic–pituitary–testicular axis, and comes with the risk of serious side effects, such as erythrocytosis, lipid metabolism disturbance, infertility and others.^8^ In addition, as physiological requirements of testosterone vary in individuals,^9^ it is difficult for exogenous testosterone supplementation to meet the requirements of individualized treatment. Therefore, it becomes necessary to explore a new therapy for testosterone supplementation in a physiological pattern.

Theoretically, because they are the primary source of testosterone,^10^ Leydig cell (LC) transplantation is a physiological therapy which could provide long-lasting delivery of testosterone. However, LCs account for only about 2–4% of the total testicular cell population in adult human testes.^11^ Moreover, LCs are terminally differentiated cells with no proliferation capacity.^10^ Therefore, it is difficult to apply LC transplantation therapy directly in clinic. LCs arise from undifferentiated stem Leydig cells (SLCs), which first develop in the neonatal testicular interstitium.^12, 13, 14, 15^ SLCs undergo phased transitions through progenitor and immature stages, and ultimately to terminally differentiated adult LCs stage.^16^ Fully grown males maintain a population of SLCs residing in the peritubular layer^17^ and/or testicular vasculature.^12, 18^ Adult SLCs, which are normally dormant, can regenerate new LCs to replace senescent or injured ones, thereby contributing to the maintenance of testicular homeostasis.^18, 19^ Recent studies on rodent SLCs have demonstrated that transplanted SLCs could replace the chemically disrupted or senescent LCs for testosterone production,^13, 14, 20^ indicating that SLC transplantation is a promising therapy for hypogonadism. In 2014, Landreh *et al.*^21^ revealed that human testicular peritubular cells expressed SLC markers (PDGFR*α*, LIFR) and that their steroidogenic potential could be activated in culture, which indicated the existence of SLCs in adult human testes. Therefore, for clinical application, it is essential to identify, isolate and *in vitro* expand human SLCs.

Here, we evaluate the use of p75 as a cell surface marker for identifying and isolating SLCs from human testes, and also demonstrate the stem cell characteristics of p75^+^ cells. We then demonstrate that transplanted p75^+^ SLCs can restore testosterone production and promote the recovery of spermatogenesis in EDS-treated rats *in vivo*. Thus, this study may provide a new strategy for the treatment of hypogonadism.

## Results

### The identification and isolation of p75^+^ cells from adult human testes

**Figure 1:**
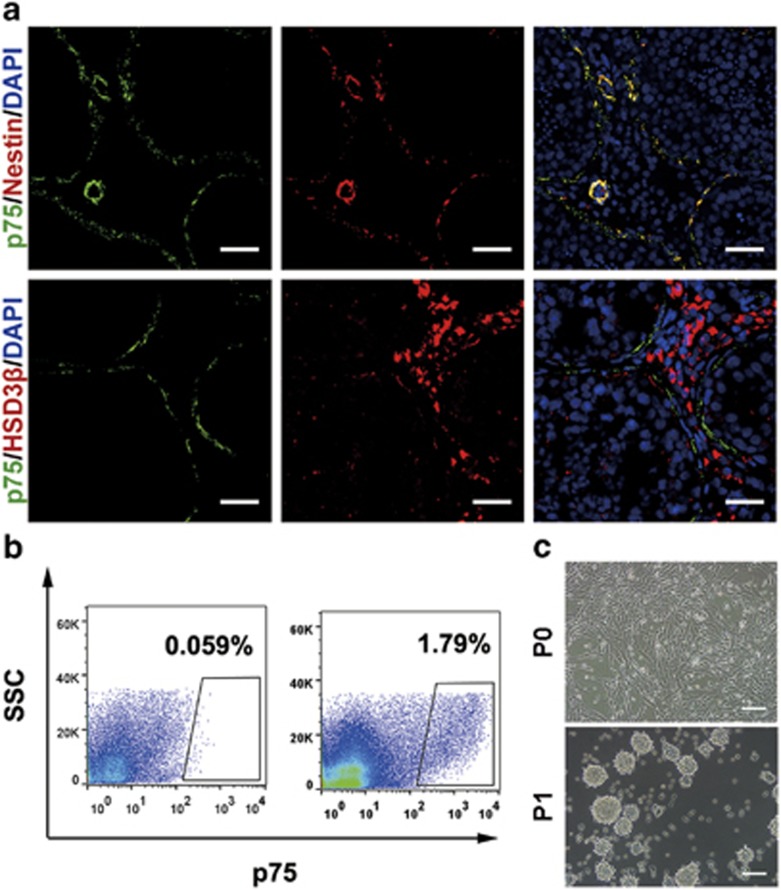
The identification and isolation of p75^+^ cells from adult human testes. (**a**) p75^+^ cells abundantly expressed nestin but only negligibly expressed HSD3*β*. Nuclei were counterstained with 4,6-diamidino-2-phenylindole (DAPI, blue). Scale bar=100 *μ*m. (**b**) Flow cytometry was used to isolate p75^+^ cells from human adult testes. The left scatter diagram shows the isotype controls and the right one shows the stained samples. (**c**) Phase-contrast micrographs of p75^+^ cells cultured in the serum-free expansion medium. Scale bar=100 *μ*m

Our previous study showed that testes-derived nestin^+^ SLCs also highly expressed CD51 and p75 in postnatal 7-day-old mice.^14^ We further confirmed that the prospectively isolated CD51^+^ cells from the testes of adult mice have SLC properties.^20^ To evaluate whether CD51 or p75 could be a putative marker specific for human SLCs, we detected the expression pattern of CD51 and p75 in human adult testes by immunostaining. Unexpectedly, HSD3*β*^+^ mature LCs expressed CD51 (Supplementary Figure 2), indicating that CD51 might not be the proper candidate for the identification and isolation of human SLCs. On the other hand, immunostaining indicated p75^+^ cells expressed known SLC marker nestin,^12, 14^ and negligibly expressed mature LC lineage marker HSD3*β* (Figure 1a). These results suggest that p75 may serve as a putative surface marker for human SLC identification and isolation. Subsequently, we isolated p75^+^ cells from human adult testes by flow cytometry (Figure 1b). The sorted p75^+^ cells were seeded in specific serum-free expansion medium. After 1 day of culture, most cells adhered to the plastic wells. When adherent cells had propagated to 80% confluence, we dissociated these cells using collagenase type IV and transferred them to a new plate for further expansion. The p75^+^ cells formed small spheres, which subsequently became floating spheres and showed proliferation ability (Figure 1c).

### The proliferation and self-renewal capacity of p75^+^ cells

**Figure 2:**
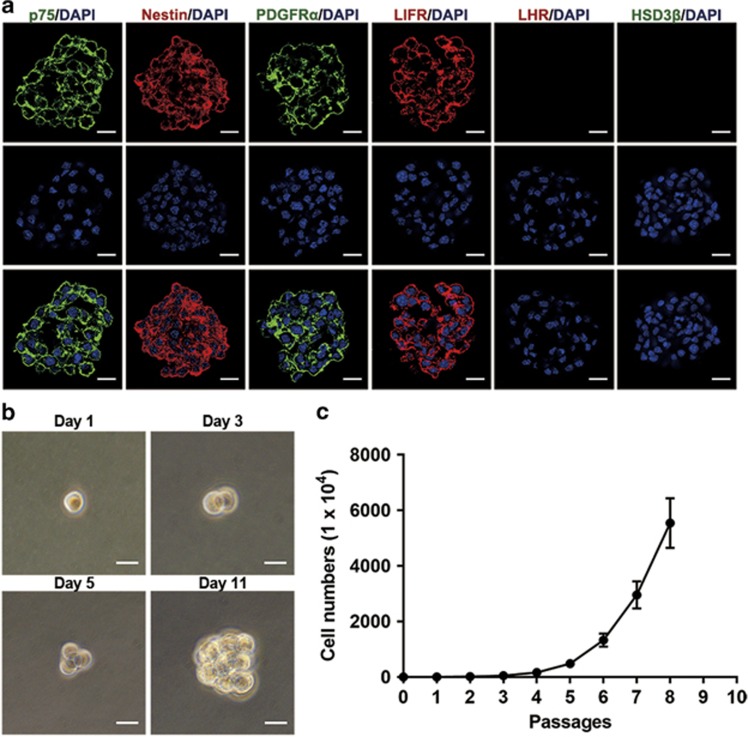
The proliferation and self-renewal capacity of p75^+^ cells. (**a**) Immunostaining showed that cultured spheres of p75^+^ cells maintained the expression of p75, nestin, PDGFR*α* and LIFR, but showed only negligible expression of LHR and HSD3*β*. Nuclei were counterstained with DAPI (blue). Scale bar=25 *μ*m. (**b**) Representative images showing the clonal cytosphere forming from a single cell. Scale bar=25 *μ*m. (**c**) Growth characteristics of p75^+^ cells cultured *in vitro*. The primary cell number was 1 × 10^4^. Data are expressed as the mean±S.D. (*n=6*)

The cytospheres of cultured p75^+^ cells at Passage 3 were collected to investigate the expression of reported SLC lineage markers by immunostaining. Interestingly, most cells within spheres expressed SLC lineage markers p75, PDGFR*α*,^13^ nestin^14^ and LIFR,^14^ but negligibly expressed LC lineage makers LHR and HSD3*β* (Figure 2a). This indicated that cultured p75^+^ cells maintained their SLC identity. To further investigate their self-renewal capacity, we carried out single-cell sphere formation assays in which single-cell suspensions derived from P1 cytospheres were seeded into 96-well plates. Seeded single cells divided and formed spheres after 11 days of culture (Figure 2b). The cytosphere with a diameter equal to or greater than 50 *μ*m in a given well was counted as one clone. The clonogenic efficiency of p75^+^ cells was 12.6±4.4%. To assess the *in vitro* expansion capacity of the p75^+^ cells, the cytospheres were enzymatically disassociated into single cells by collagenase IV for cell counting before each passage. Importantly, the p75^+^ cells could continuously proliferate for at least eight passages and expand by about 5000 times (Figure 2c). Taken together, these results demonstrate that the p75^+^ cells can expand *in vitro* and have clonogenic self-renewal capacity.

### Multi-lineage *in vitro* differentiation capacity of the p75^+^ cells

**Figure 3:**
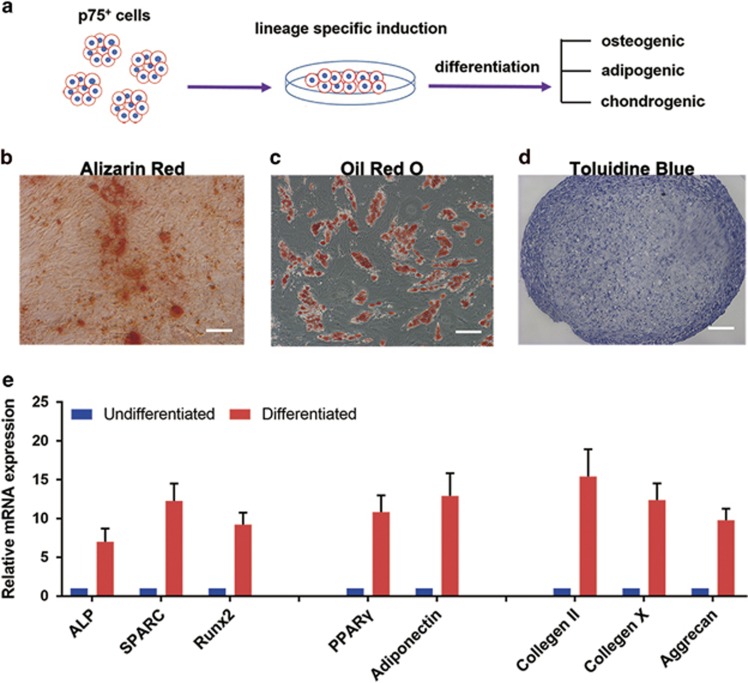
Multi-lineage *in vitro* differentiation capacity of p75^+^ cells. (**a**) Schematic of the experimental procedure used for inducing differentiation. Representative micrographs of histological staining showing p75^+^ cells differentiated into osteocytes (alizarin red) (**b**), adipocytes (oil red O) (**c**) and chondrocytes (toluidine blue) (**d**), scale bar=100 *μ*m. (**e**) Differentiated p75^+^ cells (differentiated) were examined by qRT-PCR analysis for expression of osteocyte-(ALP, SPARC and Runx2), adipocyte-(adiponectin and PPAR*γ*) and chondrocyte-(collagen II, collagen X and aggrecan) specific markers. Expression levels of each gene were compared with undifferentiated p75^+^ cells (undifferentiated). Data are expressed as the mean±S.D. (*n=3*)

To verify their plasticity, we cultured the p75^+^ cells under conditions known to favor osteogenic, adipogenic or chondrogenic differentiation (Figure 3a). After induction in osteogenic medium for 2 weeks, the p75^+^ cells differentiated into osteocytes with mineralized nodules, as shown by alizarin red staining (Figure 3b). Quantitative real-time PCR (qRT-PCR) analysis further confirmed the upregulated expression of osteocyte-specific transcripts encoding ALP, SPARC and Runx2 (Figure 3e). After 3 weeks of culture in adipogenic medium, adipogenic differentiation was confirmed by the presence of intracellular oil red O-positive lipid vacuoles (Figure 3c) and increased mRNA levels of the adipogenic markers PPAR*γ* and Adiponectin (Figure 3e). After 4 weeks of culture in chondrogenic medium, chondrogenic differentiation was identified by toluidine blue-positive cartilage-like cells and cartilage-like cavities (Figure 3d), and confirmed by significant upregulations of transcripts encoding the chondrogenic markers Collagen II, Collagen X and Aggrecan (Figure 3e). These results demonstrate that human testes-derived p75^+^ cells have multi-lineage differentiation potential *in vitro*.

### LC lineage differentiation potential of the p75^+^ cells

**Figure 4:**
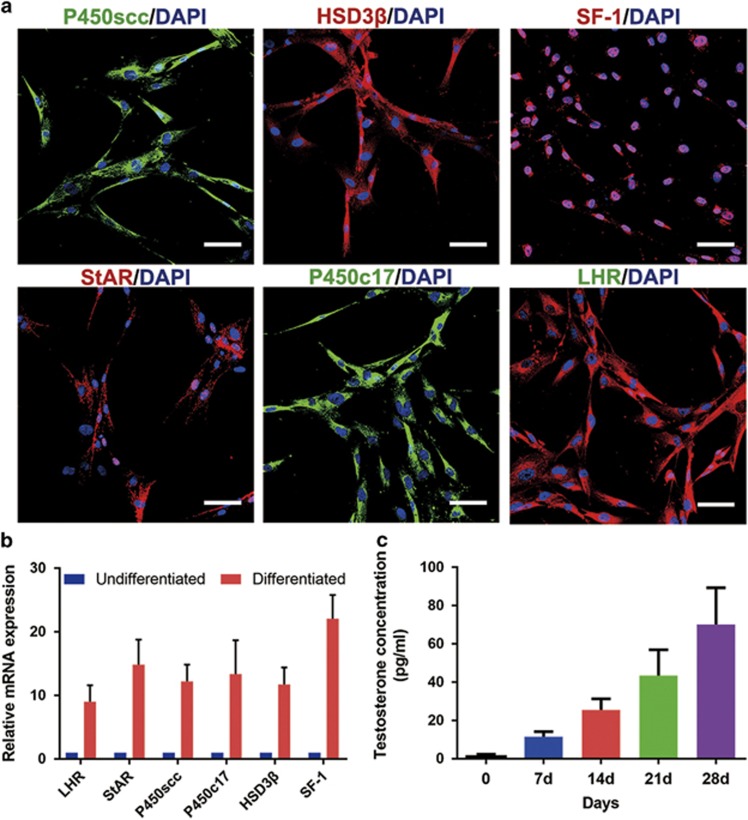
LC lineage differentiation potential of the p75^+^ cells. (**a**) After steroidogenic differentiation, immunostaining shows that the p75^+^ cells clearly express LC lineage-specific markers. The representative images show the expression of P450scc, HSD3*β*, SF-1, StAR, P450c17 and LHR in these cells. Scale bar=100 *μ*m. (**b**) Differentiated p75^+^ cells (Differentiated) were examined by qRT-PCR analysis for the expression of LC-specific markers HSD3*β*, P450scc, LHR, SF-1, StAR and P450c17. Expression levels of each gene were compared with undifferentiated p75^+^ cells (undifferentiated). Data are expressed as the mean±S.D. (*n=3*). (**c**) Testosterone production progressively increased with time during culture of the isolated cells in steroidogenic medium. Data are expressed as the mean±S.D.; six samples in three independent experiments

To determine the LC lineage differentiation potential *in vitro*, we cultured the p75^+^ cells in fresh differentiation-inducing medium. The medium, which contains luteinizing hormone (LH), insulin-like growth factor 1 (IGF-1), thyroid hormone (T3) and platelet-derived growth factor BB (PDGF-BB), was reported to promote the differentiation and survival of rodent SLCs.^13^ Indeed, after 28 days of induction, immunostaining showed that most of the differentiated cells expressed the LC lineage markers cytochrome P450 cholesterol side chain cleavage (P450scc), hydroxysteroid dehydrogenase-3*β* (HSD3*β*), steroidogenic factor 1 (SF-1), steroidogenic acute regulatory protein (StAR), cytochrome P45017A1 (P450c17) and luteinizing hormone receptor (LHR) (Figure 4a). Up-regulation of these LC lineage markers was further confirmed by qRT-PCR analysis (Figure 4b). Correspondingly, culture supernatants were harvested for quantitative testosterone determination at the indicated time points. More importantly, testosterone production of the p75^+^ cells gradually increased after induction (Figure 4c), which was consistent with the up-regulation of LC lineage markers. Taken together, these observations clearly suggest that the p75^+^ cells can differentiate into testosterone-producing LCs *in vitro*.

### Transplanted p75^+^ cells developed into functional LCs to replace the chemically disrupted LCs for testosterone production

**Figure 5:**
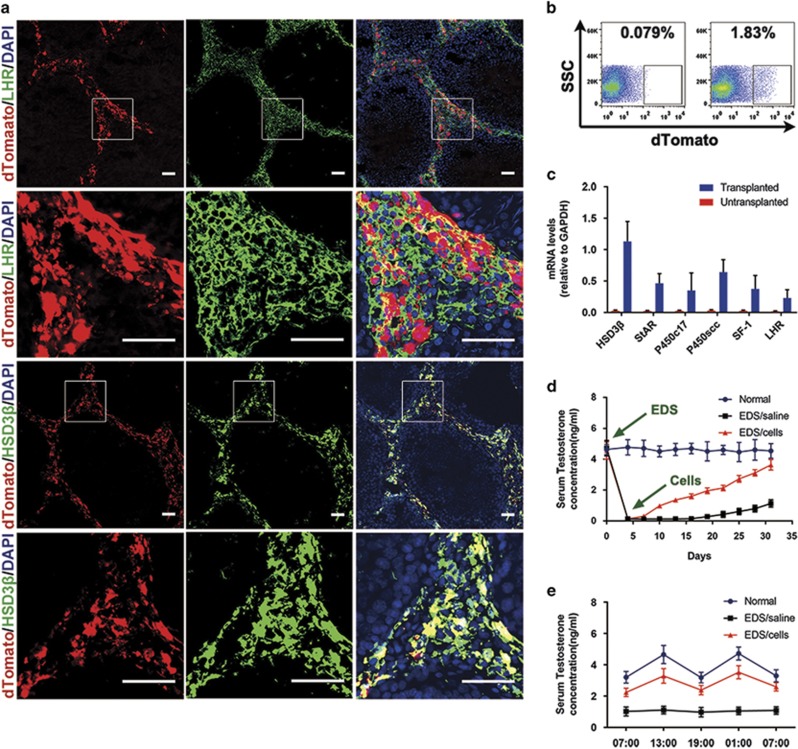
Transplanted p75^+^ cells developed into functional LCs to replace the chemically disrupted LCs for testosterone production. (**a**) Immunostaining shows the accumulation of cells positive for dTomato (red) and HSD3*β* (green) or LHR (green) in the testicular interstitia of EDS-treated rats at 21 days after the transplantation. The bottom panels show higher magnifications of the regions inside the dotted boxes of the lower-magnification images. Scale bar=100 *μ*m. (**b**) Fluorescence-activated cell sorting was used to isolate dTomato-positive cells from recipient testes. The scatter diagram on the left shows the testes of EDS/saline treated controls and that on the right shows the EDS/cells treated testes. (**c**) The mRNA expression of LC-specific markers in the sorted dTomato^+^ cells (defined as transplanted) was analyzed by qRT-PCR. Expression levels of each gene were compared to untransplanted dTomato-labeled p75^+^ cells (defined as untransplanted). (**d**) The serum testosterone levels were measured at the indicated time points in each group. The serum testosterone levels of the p75^+^ cell treated group were significantly higher compared to the EDS-treated group after cell transplantation. (**e**) Consecutive serum testosterone measurements indicated that the serum testosterone levels in EDS/cells treated rats exhibited a pulsatile biorhythm similar to (but slightly lower than) that found in the normal group. Groups: Normal, 2-month-old rats that received saline injections; EDS/saline, rats that were treated with EDS and then injected with saline 4 days later; EDS/cells, rats that were treated with EDS and then injected with dTomato-labeled p75+ cells 4 days later. Data are expressed as the mean±S.D. (*n=6*)

To trace the transplanted cells *in vivo*, the p75^+^ cells were infected with lentivirus expressing the red fluorescent protein variant dTomato. The infected cells stably expressed dTomato and proliferated as floating clonal cytospheres (Supplementary Figure 3). EDS is an alkylating agent which has selective pro-apoptotic effects on LCs.^22^ Two-month-old male rats were treated with EDS to establish LC-disrupted testosterone-deficient models. Four days after EDS treatment, serum testosterone levels of experimental rats decreased to undetectable levels and no HSD3*β*-positive cells were observed in testes by immunostaining (data not shown), which confirmed that the endogenous LCs had been completely eliminated. To further investigate the *in vivo* differentiation potential of the p75^+^ cells, we transplanted the dTomato-labeled cells into the testes of EDS-treated male rats (Supplementary Figure 1). At day 25 (21 days post-transplantation), the testes were harvested for histological analysis. Immunostaining indicated that the dTomato-labeled cells colonized the testicular interstitial (Figure 5a). Moreover, most of the dTomato-labeled cells expressed LC lineage markers LHR and HSD3*β* (Figure 5a), which indicated that transplanted p75^+^ cells might have differentiated into LCs. To further confirm the *in vivo* LC differentiation of the p75^+^ cells, the testes of recipient rats were disassociated into single cells by collagenase IV to isolate dTomato^+^ cells by flow cytometry (Figure 5b). qRT-PCR analysis demonstrated that the sorted dTomato^+^ cells expressed LC lineage markers HSD3*β*, StAR, P450c17, P450scc, SF-1 and LHR (Figure 5c). Meanwhile, some sorted dTomato^+^ cells were seeded at a density of 1 × 10^5^ cells/ml in DMEM/F12 supplemented with 10 ng/ml LH and 1 *μ*g/ml 25-hydroxycholesterol for 24 h and culture supernatants were collected for testosterone quantitative determination. The testosterone concentration of the supernatants was 4.55±0.84 ng/ml. These observations clearly suggest that the p75^+^ cells can differentiate into testosterone-producing LCs *in vivo*.

To evaluate the therapeutic effects of p75^+^ cell transplantation on EDS-treated male rats, sera were harvested for quantitative testosterone determination. p75^+^ cell transplantation significantly increased the serum testosterone levels in EDS-treated rats. Specifically, the serum testosterone levels of the cell-treated group progressively increased from 0.98±0.15 ng/ml at day 10 to 3.64±0.34 ng/ml at day 31, compared with the control group, in which levels went from undetectable at day 10 to 1.13±0.25 ng/ml at day 31 (*P*<0.01; Figure 5d). To test whether the cell-treated rats showed a diurnal testosterone rhythm, we collected blood samples every 6 h from 0700 hours at day 29 to 0700 hours at day 30 for quantitative testosterone determination. Notably, the serum testosterone levels of cell-transplanted rats exhibited a pulsatile circadian rhythm, which was similar to saline-injected normal rats (Figure 5e).

Taken together, these findings reveal that p75^+^ cells can differentiate into functional LCs *in vivo* to replace chemically disrupted LCs and secrete testosterone in a physiological pattern.

### p75^+^ cell transplantation promoted the recovery of spermatogenesis and reproductive organ weights in EDS-treated rats

**Figure 6:**
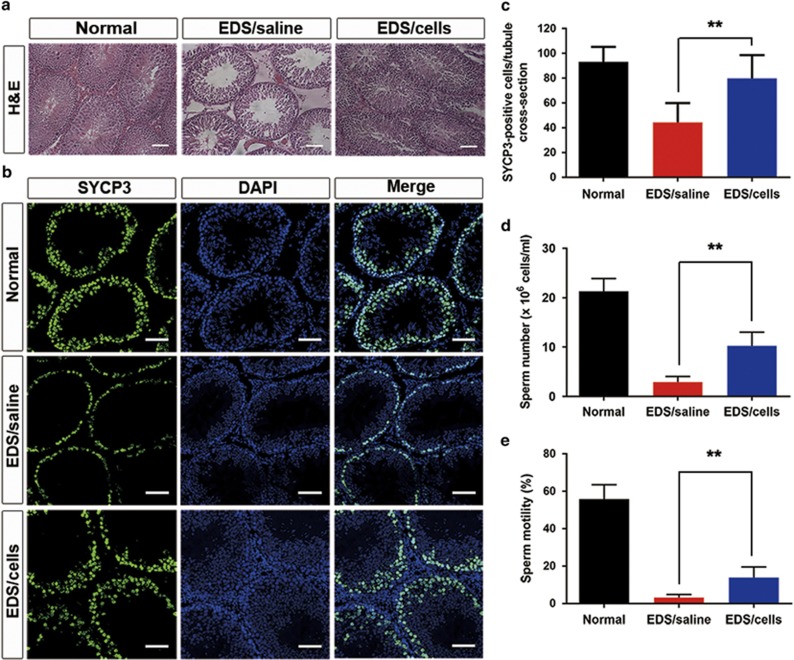
p75^+^ cell transplantation accelerated the recovery of spermatogenesis in EDS-treated rats. (**a**) H&E staining shows the structures of the testes in experimental rats. Scale bar=100 *μ*m. (**b**) Immunostaining showing SYCP3-positive (green) cells in seminiferous tubules. Scale bar=100 *μ*m. (**c**) Quantitative analysis of SYCP3-positive cells in seminiferous tubules. Sperm numbers (**d**) and sperm motilities (**e**) were analyzed in each group. Data are expressed as the mean±S.D. (*n*=6); **P<0.01

Intratesticular testosterone which directly depends on the function of endogenous LCs is crucial for spermatogenesis.^23^ To evaluate the impact of p75^+^ cell transplantation on spermatogenesis, we collected testes for histological analysis at day 25 (21 days post-transplantation). Besides Sertoli cells, seminiferous tubules chiefly consist of various types of spermatogenic cells. Thus, the thickness of seminiferous tubules primarily depends on the quantity of spermatogenic cells.^24^ Accordingly, hematoxylin and eosin (H&E) staining was used to assess the development of spermatogenic cells in general. Remarkably, the internal diameters of the seminiferous tubules in the cell-treated group were thicker compared with the EDS-treated group (Figure 6a). Synaptonemal complex protein 3 (SYCP3) is a meiosis-specific marker^25^ which was widely used to evaluate spermatogenesis. Correspondingly, immunostaining demonstrated that the quantity of SYCP3-positve cells per seminiferous tubule in the cells treated group was significantly higher than in the EDS-treated controls (Figures 6b and c), indicating that p75^+^ cell transplantation increased the amount of spermatogenic cells in the meiotic stage. Soon after their formation in the seminiferous tubules, spermatozoa enter the epididymis, where they undergo maturation processes necessary for them to acquire motility.^26^ Thus, low testicular testosterone levels would affect sperm quality finally.^1, 27^ To determine the influence of p75^+^ cell transplantation on sperm quality, epididymides were harvested for semen analysis at day 32 (28 days post-transplantation). Notably, the sperm number and sperm motility in the epididymides significantly increased in the cells treated group compared with in the EDS-treated controls (Figures 6d and e). All these findings indicate that p75^+^ cell transplantation facilitates spermatogenesis recovery in EDS-treated male rats.

As testosterone and its derivative dihydrotestosterone are essential for maintenance of the mass of reproductive organs,^28^ testosterone deficiency can cause atrophy of reproductive organs.^1^ To monitor the impact of p75^+^ cell transplantation on reproductive organs, we weighed the testes, epididymides, prostates and seminal vesicles at day 32 (28 days post-transplantation). Notably, p75^+^ cell transplantation significantly increased the weights of the testes, prostates, seminal vesicles and epididymides of EDS-treated rats (Supplementary Figure 4).

## Discussion

In this study, we sought to identify and isolate human SLCs for cell replacement therapy for male hypogonadism. We identified that p75^+^ cells from adult human testes possessed similar properties to rodent SLC populations, and that transplanted human p75^+^ SLCs could differentiate into functional LCs to replace the chemically disrupted LCs and partially restore testosterone production in EDS-treated male rats. This study provides new insights into the clinical application of human SLCs for hypogonadism.

Previously, we demonstrated^14^ that nestin^+^ cells, which were isolated from the testes of Nestin-GFP transgenic mice, have the capacities for continuous expansion and multi-potential differentiation *in vitro*. When transplanted into the testes of EDS-treated rats, these cells could differentiate into testosterone-producing LCs. Therefore, the testes-derived nestin^+^ cells are de facto SLCs. However, nestin is an intracellular cytoskeletal protein,^29^ which means that the identification of nestin in tissues of nontransgenic animals and humans would require cell permeabilization, excluding its application for human cell isolation. Therefore, it is essential to exploit surface markers for the isolation and *in vivo* expansion of human SLCs. However, by using flow cytometry, we have revealed that in Nestin-GFP transgenic mice most nestin^+^ SLCs expressed CD51 and p75 (respectively, 93.53%±3.10% and 74.73%±1.89%).^14^ Nonetheless, the expression patterns of p75 and CD51 in human adult testes require further clarification. CD51, also known as integrin alpha-V, is reported to be expressed in nestin^+^ colorectal cancer stem cells.^30^ CD51 was also used to isolate nestin^+^ mesenchymal stem cell (MSCs) in human fetal bone marrow.^31^ However, CD51 cannot be used to identify adult nestin^+^ MSCs, as both osteoblasts^32^ and megakaryocytes^33^ express CD51 in human adult marrow. Previously, we have demonstrated that CD51 was the surface marker specific for nestin^+^ SLCs in mouse testes.^20^ Here, we found that mature LCs also expressed CD51 in adult human testes, which indicates that CD51 might not be a candidate for the identification and isolation of human SLCs. p75, also known as p75NTR and CD271, is the low affinity receptor of neurotrophin. While playing a great role in the survival, migration and differentiation of stem cells, it labels various neural stem cells, neural crest stem cells and some MSC populations.^34^ Thereby, p75 may be a common surface marker specific for nestin^+^ stem cells. Correspondingly, we investigated the expression pattern of p75 in adult human testes by immunostaining. p75^+^ cells expressed SLC specific marker nestin, but not LC lineage marker HSD3*β*, which indicates that p75 might be a potential surface marker specific for human nestin^+^ SLCs.

Importantly, on the basis of p75 expression, we established for the first time a culture system for the *in vitro* expansion of human SLC. We sorted p75^+^ cells from adult human testes by flow cytometry and cultured them in a serum-free medium. The p75^+^ cells exhibited the capacities of clonogenic self-renewal and multi-potential differentiation into mesenchymal cell lineages and functional LCs *in vitro*. Interestingly, transplanted human p75^+^ SLCs differentiated into testosterone-producing LCs, which expressed LC lineage markers at both RNA and protein levels. Furthermore, when cultured *in vitro* with LH and 25-hydroxycholesterol, the transplanted human p75^+^ SLCs could produce testosterone. These findings clearly suggest that p75 can served as a novel surface marker to identify and isolate human SLCs.

As dysfunction of senescent LCs is the primary cause of hypogonadism, theoretically, stem cell transplantation to replace the endogenous senescent or disrupted LCs may be a promising therapy. Recent studies indicate that rodent SLCs can differentiate into LCs *in vivo* to replace the senescent and chemically disrupted LCs for testosterone production.^13, 14, 20^ Therefore, we further explored the therapeutic effects of human p75^+^ SLC transplantation on EDS-treated male rats. Notably, the serum testosterone levels of recipient rats were much higher than EDS-treated control rats and exhibited circadian rhythm, which indicated that transplanted human p75^+^ SLCs could secrete testosterone in a physiological pattern. Testosterone is essential for spermatozoa development^35^ and reproductive organ mass,^28^ while patients with hypogonadism often suffer spermatogenesis dysfunction and atrophy of genital organs.^1, 6^ Thus, we investigated the spermatogenesis and reproductive organ weights of the experimental rats. Importantly, human p75^+^ SLC transplantation facilitated the recovery of spermatogenesis and reproductive organ weights in EDS-treated rats. These results indicate that transplanted p75^+^ SLCs could replace the disrupted LCs to restore testosterone production in testosterone-deficient male rats.

In summary, we have identified that p75 is a novel surface marker specific for the identification and isolation of human SLCs, and we have investigated the therapeutic effects of p75^+^ human SLC transplantation on EDS-treated LC-disrupted male rats. Our findings provide a new approach for further clinical application of human SLCs for male hypogonadism.

## Materials and methods

### Human testes samples and animals

As previously described,^21^ human testes tissue samples were obtained from two brain-dead donors (18 and 19 years old, respectively) and four patients affected by obstructive azoospermia (23, 25, 28 and 32 years old, respectively). Written informed consents were obtained from the patients or from the parents of the brain-dead donors. Ethical approval for this study was obtained from the ethics committee of Zhongshan Medical School (Sun Yat-sen University).

Two-month-old male Sprague-Dawley rats were obtained from the experimental animal center of Sun Yat-sen University. All rats were kept under controlled temperature (24 °C±1 °C) and relative humidity (50–60%) conditions, with a 12-h light/12-h dark cycle, and free access to standard rodent diet and drinking water. All surgical procedures and postoperative care were approved by the Institutional Animal Care and Use Committee of Sun Yat-sen University.

### Isolation and culture of p75^+^ cells from human testes

Human primary p75^+^ cells were isolated from the testes tissues of six donors, as previously described^14^ with minor modifications. In brief, the human testes tissue was enzymatically disassociated by 1 mg/ml collagenase type IV (Sigma, St. Louis, MO, USA) at 37 °C for 15 min, filtered through a 45-*μ*m filter, and centrifuged at 1500 rpm for 5 min at room temperature. The pellet was washed with phosphate-buffered saline (PBS) and then incubated with anti-human p75 and isotype control antibodies (BD Bioscience, Franklin Lakes, NJ, USA) in the dark at room temperature for 15 min. The p75^+^ cells were enriched by flow cytometry using an Influx Cell Sorter (BD Bioscience, Franklin Lakes, NJ, USA).

For the culture of p75^+^ cells, we used a previously published expansion medium^14^ with some modifications. Briefly, Dulbecco’s modified Eagle’s medium (DMEM/F12) (1:1; Gibco, Waltham, MA, USA) was mixed 1:1 with neurobasal medium (Gibco), and then supplemented with 15% chicken embryo extract (Us Biologicals, Swampscott, MA, USA), 1% nonessential amino acids (Hyclone, Waltham, MA, USA), 1% N2, 2% B27 supplements, 0.1 mM *β*-mercaptoethanol (Gibco), 20 ng/ml recombinant fibroblast growth factor-basic, epidermal growth factor, platelet-derived growth factor BB and oncostatin M (Pepro Tech, Rocky Hill, NJ, USA). The cultures were incubated at 34 °C in a humidified 5% CO_2_ water-jacketed incubator. The medium was changed every 3 days.

### RNA isolation and qRT-PCR

Total RNA was extracted using an RNeasy mini kit (Qiagen, Dusseldorf, German) according to the manufacturer’s protocol. Reverse transcription was performed using murine leukemia virus reverse transcriptase and oligo-dT primers (Fermentas, Vilnius, Lithuania). qRT-PCR was performed using the Thunderbird SYBR qPCR Mix (Toyabo, Osaka, Japan) according to the manufacturer’s instructions. Signals were detected using a Light Cycler 480 Detection System (Roche, Basel, Switzerland). The sequences of the primers used are listed in Supplementary Table 1.

### *In vitro* differentiation of p75^+^ cells

For osteogenic differentiation, p75^+^ cells were plated in high-glucose DMEM (H-DMEM; Gibco) containing 20% fetal bovine serum (FBS) (Hyclone), 100 *μ*g/ml ascorbic acid, 100 nM dexamethasone, 10 mM *β*-glycerophosphate (Sigma, St. Louis, MO, USA), and 100 IU/ml penicillin/streptomycin (Gibco). The cells were cultured for 2 weeks with feedings every 3 days. Osteogenic differentiation was investigated by alizarin red staining and qRT-PCR for osteogenic makers.

For adipogenic differentiation, the cells were induced in H-DMEM supplemented with 10% FBS, 100 nM dexamethasone, 10 *μ*g/ml insulin, 0.2 mM indomethacin, 0.5 mM 3-isobutyl-1-methylxanthine (Sigma) and 100  IU/ml penicillin–streptomycin. After 3 weeks, adipogenic differentiation was confirmed by oil red O staining and qRT-PCR for adipogenic makers.

For chondrogenic differentiation, the cells were induced using a cell pellet culture system. In brief, the p75^+^ cells were suspended in a 15-ml conical tube containing 2 ml induction medium consisting of H-DMEM supplemented with 3% FBS, 1% insulin-transferrin-sodium selenite (ITS; Gibco), 1 mm pyruvate (Sigma), and 10 ng/ml transforming growth factor-*β*3 (PeproTech). The cells were fed every 3 days, and after 4 weeks of culture, chondrogenic differentiation was investigated by toluidine blue staining and qRT-PCR for chondrogenic makers.

For LC lineage differentiation, 1 × 10^4^ p75^+^ cells at Passage 3 were disassociated into single cells and plated in a 24-well plate with 0.5 ml fresh differentiation-inducing medium^13, 14^ containing phenol red-free DMEM/F12, 2% FBS, 1% ITS, 1 nM thyroid hormone, 1 ng/ml luteinizing hormone (Sigma), 10 ng/ml PDGF-BB and 70 ng/ml insulin-like growth factor 1 (PeproTech) for 4 weeks. Steroidogenic differentiation was confirmed by immunostaining and qRT-PCR for LC lineage-specific markers.

### Testosterone concentration assay

The testosterone concentrations in cell culture supernatants and rat sera were measured using a commercially available ELISA kit (R&D Systems, Minneapolis, MN, USA) according to the manufacturer’s instructions.

### The transplantation of p75^+^ cells

For labeling, p75^+^ cells were transduced with lentiviral vectors expressing the red fluorescent protein variant dTomato driven by the *EF-1α* promoter (Supplementary Figure 3). In brief, p75^+^ cells were infected by the lentivirus with 6 mg/ml of polybrene (Sigma). The medium was changed to fresh culture medium 12 h after infection. There days later, dTomato^+^ cells were purified by flow cytometry.

The rats were injected intraperitoneally with a single dose of EDS (75 mg/kg body weight) to eliminate their endogenous LCs. Four days after EDS treatment, the dTomato-labeled p75^+^ cells (~1 × 10^6^ cells in 25 *μ*l PBS) were injected into the parenchyma of the recipient testes. As a control, EDS-treated rats were subjected to testicular injection of the same volume of saline.

### Histological analysis

For immunostaining, cytospheres, human testes tissues and rat testes were fixed with 4% paraformaldehyde in PBS, dehydrated with 30% sucrose solution in PBS, and then cryo-embedded in optimal cutting temperature medium (Sakura Finetek, Tokyo, Japan). Sections of 5 *μ*m thickness were blocked by incubation in 5% BSA (Sigma) in PBS for 30 min at room temperature, and then incubated overnight with primary antibodies at 4 °C. The sections were then washed and incubated with secondary antibodies at room temperature for 30 min in the dark. Negative controls were prepared by substituting PBS for the primary antibody. Images were obtained using an LSM800 or LSM 710 confocal microscope (Zeiss, Heidenheim, Germany), and were analyzed using Image J software (National Institutes of Health, Bethesda, MD, USA). For quantitative analysis, the number of synaptonemal complex protein 3-positive cells per tubule in a testis cross-section was counted as previously described.^14^ The primary and secondary antibodies used are listed in Supplementary Table 2.

To investigate the testicular structure, rat testes were fixed with Bouin’s fixative and embedded in paraffin. Sections of 5 *μ*m thickness were stained with H&E.

### Statistical analysis

All data are presented as the mean±S.D. obtained from at least three independent experiments. Comparisons between groups were performed using a one-way analysis of variance or Student’s *t*-test. *P*<0.05 was considered statistically significant.
