# Supplementary material for: Transplanted human p75-positive stem Leydig cells replace disrupted Leydig cells for testosterone production
Source: Cell Death Dis. 2017 Oct 12;8(10):e3123–. doi: 10.1038/cddis.2017.531 (PMC5680910; doi:10.1038/cddis.2017.531)
Supplement: Supplementary Information [file cddis2017531x1.docx]

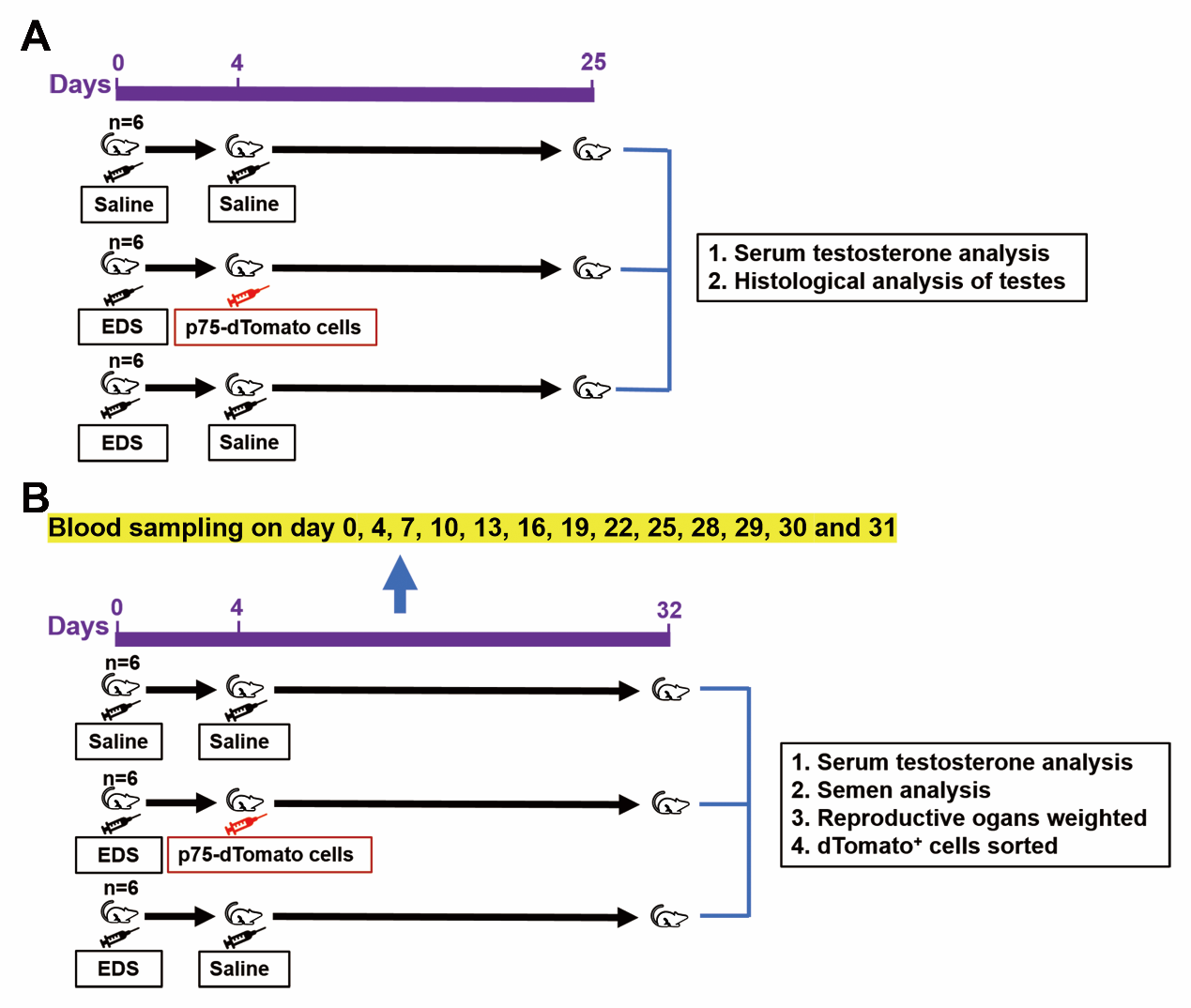


**Figure S1:** Schematic of the experimental procedure used for cell transplantation. A: Immunostaining and H&E staining analysis of the testes of experimental rats at day 25 (21 days after transplantation). B: Serum testosterone levels analysis, epididymis semen analysis, and the expression of LC lineage markers and testosterone production capacity of dTomato^+^ cells sorted from the testes of experimental rats.


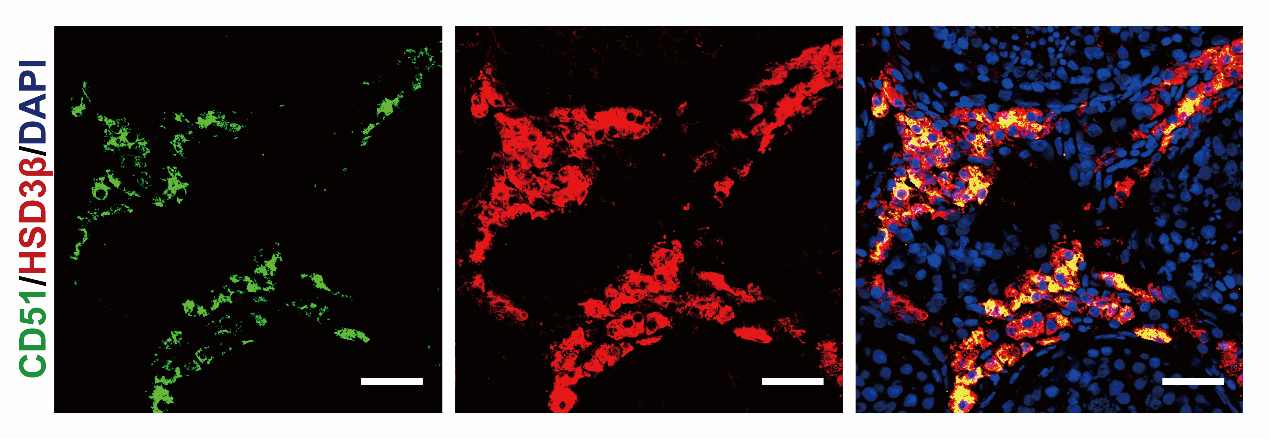


**Figure S2:** The expression pattern of CD51 in human adult testes. The CD51^+^ cells in the testicular interstitium co-expressed the LC lineage marker HSD3β. Scale bar = 100 µm.


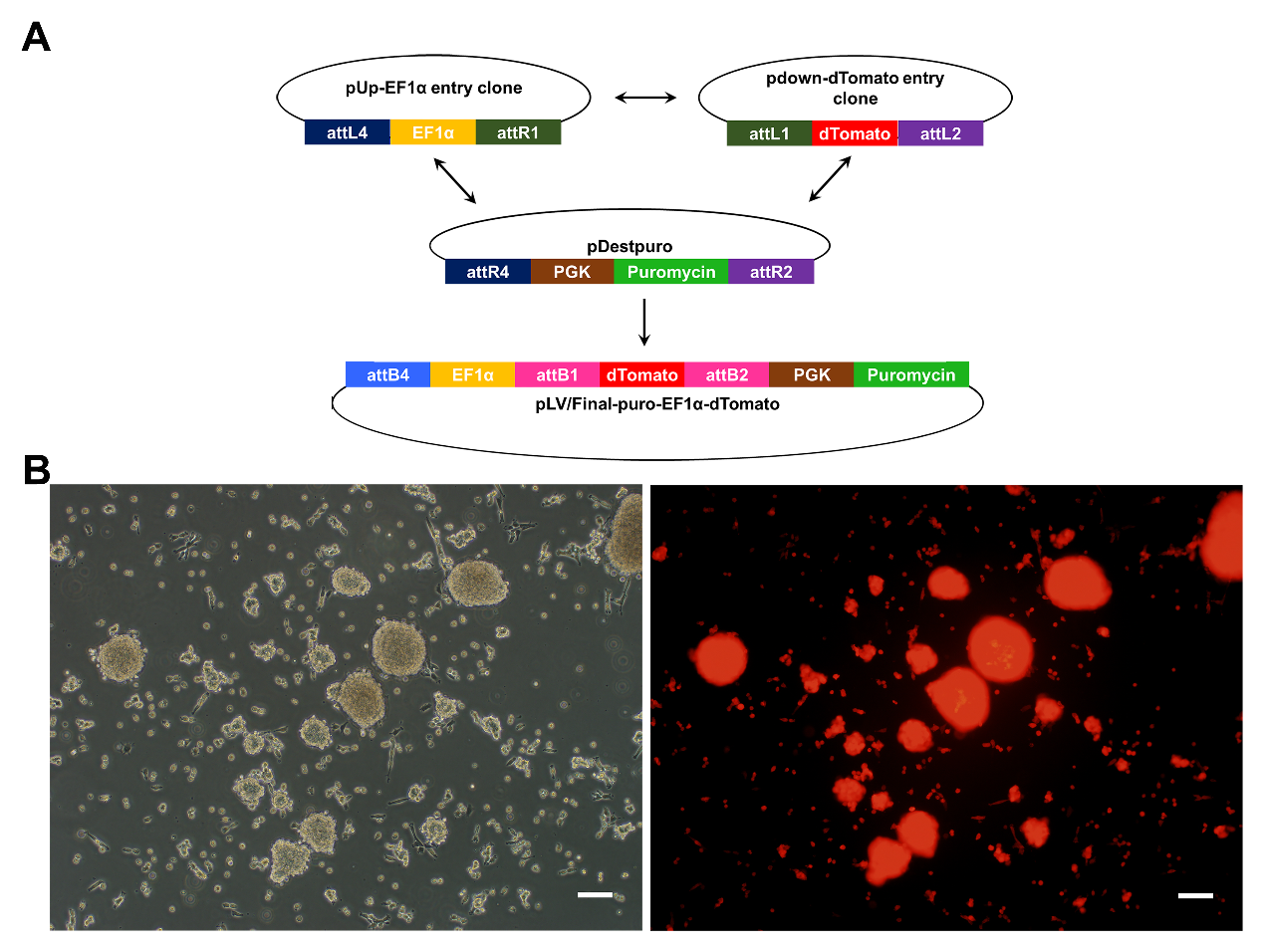


**Figure S3:** The dTomato labeling of human p75^+^ SLCs. A: Vector construction. Schematic diagram showed the construction of the pLV/Final-Puro-EF1α-dTomato vector. B: The dTomato-labeled p75^+^ cells propagated to floating clonal spheres in the serum-free expansion medium. Scale bar = 100 µm.


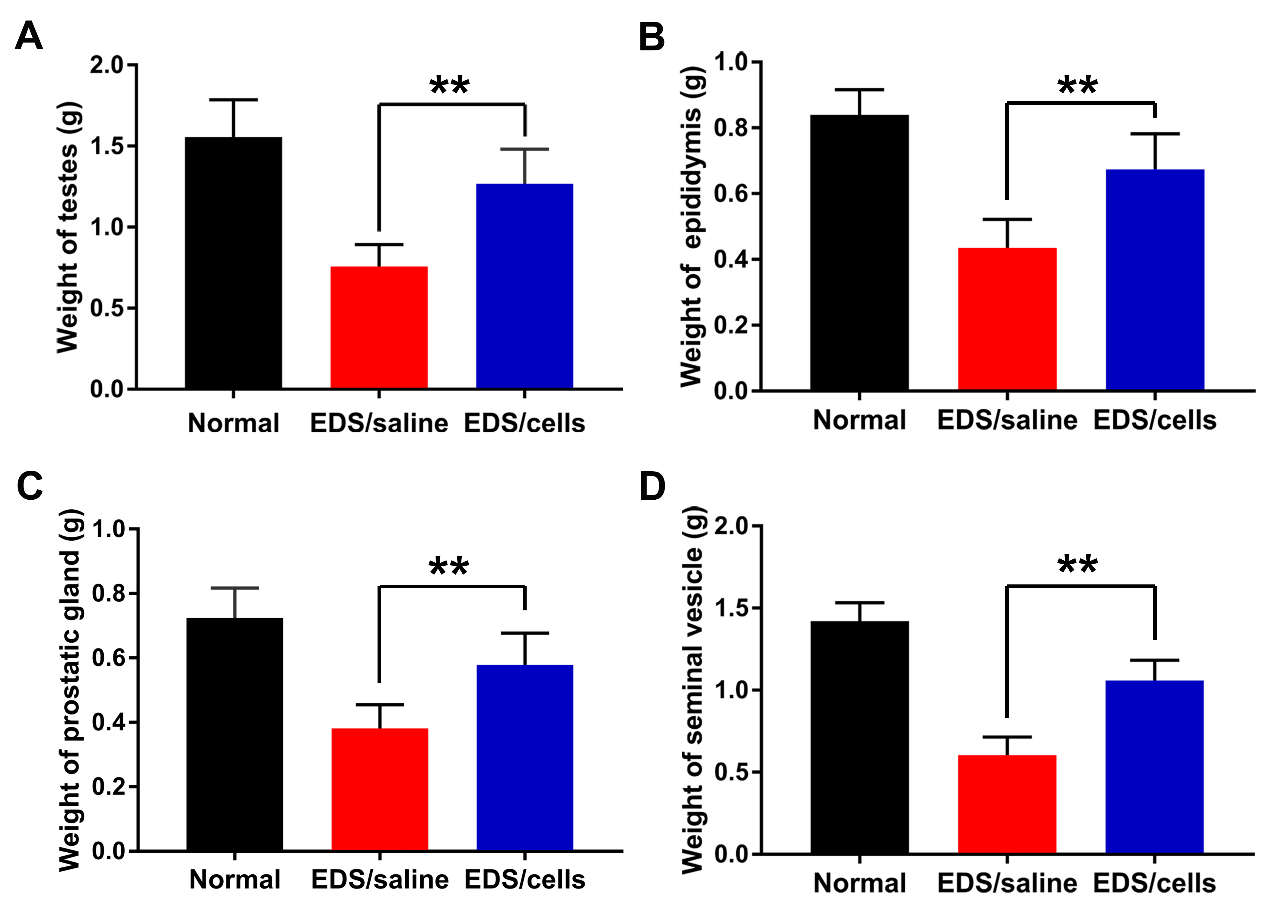


**Figure S4:** The weights of reproductive organs of each experiment rats. The reproductive organs testes (A), epididymis (B), prostatic glands (C), seminal vesicles (D) of EDS/cells treated group are heavier than EDS/saline treated group, but still lighter than normal control group. Data are expressed as the mean ± SD (*n=6*), ** *P*<0.01.

**Table S1:** Primers used to amplify transcripts during qRT-PCR analysis.

| **Gene** | **Forward Primer** | **Reverse Primer** |
| --- | --- | --- |
| collagen II  collagen X  aggrecan  adiponectin  PPARγ  ALP  SPARC  [Runx2](https://www.ncbi.nlm.nih.gov/gene/12393)  LHR  HSD3β  StAR  P450scc  P450c17  SF-1  GAPDH | TGGACGCCATGAAGGTTTTCT  GGGGCTAAGGGTGAAAGGG  GTGCCTATCAGGACAAGGTCT  CAGGCCGTGATGGCAGAGATG  TACTGTCGGTTTCAGAAATGCC  AACATCAGGGACATTGACGTG  TGCCTGATGAGACAGAGGTGGT  CCGCCTCAGTGATTTAGGGC  ATGGGACGACACTGACTTCAC  GTCTTCGGTGTCACTCACAGAG  GGGAGTGGAACCCCAATGTC  GCAGTGTCTCGGGACTTCG  TATGGCCCCATCTATTCGGTT  CCGGCTACCACTACGGACT  TGTGGGCATCAATGGATTTGG | TGGGAGCCAGATTGTCATCTC  GGTCCTCCAACTCCAGGATCA  GATGCCTTTCACCACGACTTC  GGTTTCACCGATGTCTCCCTTAG  GTCAGCGGACTCTGGATTCAG  GTATCTCGGTTTGAAGCTCTTCC  CTTCGGTTTCCTCTGCACCATC  GGGTCTGTAATCTGACTCTGTCC  GCCTGCAATTTGGTGGAAGA  CTGGTGTAGATGAAGACTGGCAC  CCAGCTCGTGAGTAATGAATGT  GGCAAAGCGGAACAGGTCA  GCGATACCCTTACGGTTGTTG  CTGCGTCTTGTCGATCTTGC  ACACCATGTATTCCGGGTCAAT |

**Table S2:** Primary and secondary antibodies used for immunostaining or flow cytometry analysis.

| **Antibodies (SPECIES, IgG TYPE)** | **DILUTION** | **DISTRIBUTOR (Cat.NO)** | |
| --- | --- | --- | --- |
| goat polyclonal to 3β-HSD(D-18)  rabbit polyclonal to LHR(H-50)  rabbit polyclonal to SF-1(H-60)  mouse polyclonal to StAR(D-2)  goat polyclonal to CYP17A(C-17)  P450scc Enzyme rabbit IgG  Rabbit anti-p75 NGF receptor IgG  Rabbit anti- Integrin alpha V antibody  Rabbit anti-PDGFRα antibody  Mouse monoclonal IgG to SYCP3(D-1)  Mouse anti-Human CD271 antibody  Goat Anti-rat IgG Alexa 594  Goat Anti-rabbit IgG Alexa 488  Goat Anti-rabbit IgG Alexa 594  Goat Anti-mouse IgG Alexa 488  Goat Anti-mouse IgG Alexa 594  Donkey Anti-goat IgG Alexa 488  Donkey Anti-goat IgG Alexa 594 | 1:100  1:150  1:150  1:100  1:150  1:200  1:100  1:100  1:100  1:200  1:100  1:1000  1:1000  1:1000  1:1000  1:1000  1:1000  1:1000 | | Santa Cruz (sc30820)  Santa Cruz (sc25828)  Santa Cruz (sc28740)  Santa Cruz (sc166821)  Santa Cruz (sc-46081)  Millipore (AB11244)  Abcam (ab8874)  Abcam (ab179475)  Millipore (07-276)  Santa cruz (sc-74569)  BD Bioscience (560326)  Invitrogen (A11007)  Invitrogen (A11008)  Invitrogen (A11037)  Invitrogen (A11001)  Invitrogen (A11032)  Invitrogen (A11055)  Invitrogen (A11058) |
